# Activation of the Human MT Complex by Motion in Depth Induced by a Moving Cast Shadow

**DOI:** 10.1371/journal.pone.0162555

**Published:** 2016-09-06

**Authors:** Narumi Katsuyama, Nobuo Usui, Masato Taira

**Affiliations:** 1 Department of Cognitive Neurobiology, Graduate School of Medical and Dental Sciences, Tokyo Medical and Dental University, Bunkyo-ku, Tokyo, Japan; 2 Center for Brain Integration Research, Tokyo Medical and Dental University, Bunkyo, Tokyo, Japan; University of Montreal, CANADA

## Abstract

A moving cast shadow is a powerful monocular depth cue for motion perception in depth. For example, when a cast shadow moves away from or toward an object in a two-dimensional plane, the object appears to move toward or away from the observer in depth, respectively, whereas the size and position of the object are constant. Although the cortical mechanisms underlying motion perception in depth by cast shadow are unknown, the human MT complex (hMT+) is likely involved in the process, as it is sensitive to motion in depth represented by binocular depth cues. In the present study, we examined this possibility by using a functional magnetic resonance imaging (fMRI) technique. First, we identified the cortical regions sensitive to the motion of a square in depth represented via binocular disparity. Consistent with previous studies, we observed significant activation in the bilateral hMT+, and defined functional regions of interest (ROIs) there. We then investigated the activity of the ROIs during observation of the following stimuli: 1) a central square that appeared to move back and forth via a moving cast shadow (mCS); 2) a segmented and scrambled cast shadow presented beside the square (sCS); and 3) no cast shadow (nCS). Participants perceived motion of the square in depth in the mCS condition only. The activity of the hMT+ was significantly higher in the mCS compared with the sCS and nCS conditions. Moreover, the hMT+ was activated equally in both hemispheres in the mCS condition, despite presentation of the cast shadow in the bottom-right quadrant of the stimulus. Perception of the square moving in depth across visual hemifields may be reflected in the bilateral activation of the hMT+. We concluded that the hMT+ is involved in motion perception in depth induced by moving cast shadow and by binocular disparity.

## Introduction

Motion perception in depth is a fundamental process for the recognition and manipulation of moving objects, and for avoidance of looming obstacles. Recent studies have revealed that the human MT complex (hMT+) may play a role in motion perception in depth. The hMT+ consists of the human MT (hMT), which is highly sensitive to two-dimensional (2D) motion, and surrounding areas that are also motion-sensitive [1–4]. Clinical studies showed that bilateral lesions including the hMT+ cause severe deficits in motion perception in three-dimensional (3D) space as well as in the 2D frontal plane, while perception of other visual properties including shape, color, and position is preserved [5–7]. Consistent with clinical observations, functional imaging studies with normal participants have revealed that the hMT+ is sensitive to motion in depth represented by binocular depth cues [8, 9]. The hMT+ is also sensitive to static binocular disparity [10–13].

The perception of motion in depth can be also induced by monocular depth cues such as optic flow, motion parallax, and relative changes in the size and position of retinal images [14]. Functional imaging studies have revealed that the hMT+ is sensitive to the inward and outward motion of the optic flow, to biological motion, and to 3D shape from motion [15–20].

**Fig 1:**
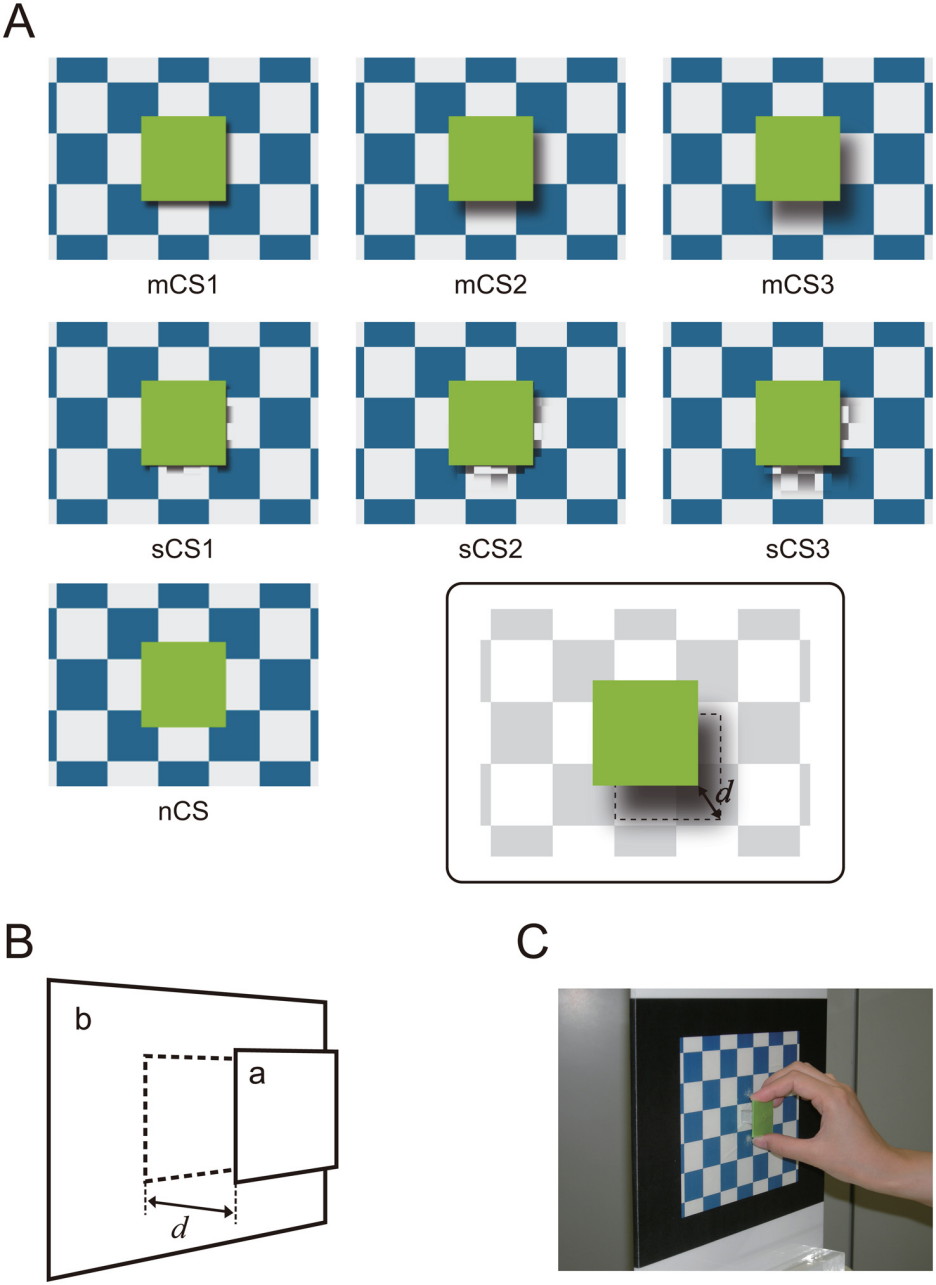
A: Examples of visual stimuli presented in the cast shadow (CS) session. Movie frames of the moving cast shadow (mCS), scrambled cast shadow (sCS), and no cast shadow (nCS) conditions are shown in the top, middle, and bottom of the figure, respectively. In the sCS condition, a segmented and temporally scrambled cast shadow was presented at the bottom-right corner of the square. In the nCS condition, no cast shadow was presented. The configurations of the central square and background were constant in all stimuli. B: Schematic diagram of the visual stimulus presented in the stereomotion localizer (SL) session. In the moving square (mSQ) condition, a central square (a) moved away from and toward a background (b) by random dot stereogram. In the static square (sSQ) condition, the square was presented at the foremost position. In the no square (nSQ) condition, only the background was presented. C: Test board used in the distance estimation test. After the scanning sessions, participants observed each of the visual stimuli that had been presented in the SL and CS sessions, and were required to manually move a square plate on the board so that the distance between the plate and background matched the perceived distance between the square and background in the stimulus.

A moving cast shadow is a powerful monocular depth cue for the perception of motion toward and away from the observer [21–24]. This is effectively demonstrated by the ‘square-over-checkerboard’ (SOC) motion illusion. In the SOC illusion, a square and its cast shadow are presented in front of a checkerboard background (Fig 1A, top). When the cast shadow moves away from or toward the square at its bottom-right corner on the background, the square appears to move toward or away from the observer in depth, whereas the size and position of the square are actually constant [22, 23]. The effect of the moving cast shadow as a depth cue in the SOC is so influential that it can override other monocular depth cues, such as changes in the relative size and position of the retinal image [22, 23]. This effect can even dominate binocular disparity within a certain range (For a demonstration, see Dr. Kersten’s laboratory website: http://gandalf.psych.umn.edu/users/kersten/kersten-lab/demos/shadows.html. Accessed 15 July 2016). Despite the strength of this phenomenon, the underlying neuronal mechanisms are unknown. A recent study has revealed that the motion in depth in the SOC illusion is perceptually comparable with that represented by binocular disparity [24]. Taken together with the observations previously described, this finding strongly suggests that the hMT+ is also involved in the perceptual process of the SOC illusion. In the present study, we examined this possibility by using a functional magnetic resonance imaging (fMRI) technique and region of interest (ROI) analyses. We first explored the cortical regions sensitive to the motion in depth represented by a binocular disparity cue. Consistent with the previous studies [8, 9], we found significant activation in the bilateral hMT+. The activations were designated as the stereomotion regions (SMRs) in the present study, and functional ROIs were defined on them. Next we examined the activity of the SMRs during observation of the stimulus that produced motion in depth by a moving cast shadow. Our results indicated that the hMT+ is activated bilaterally during observation of motion in depth induced by both moving cast shadow and binocular disparity, suggesting that the SMRs may be involved in motion perception in depth across the visual hemifields, independent of depth cues.

## Materials and Methods

### Participants

Twenty-one healthy individuals (15 male and 6 female, mean age ± SD = 24.1 ± 5.1 years) took part in this study. All were right-handed (Edinburgh Handedness Inventory score: 81.2 ± 22.1), and had normal or corrected-to-normal vision. None had a history of neurological or psychiatric diseases. All participants took part in both the fMRI and behavioral experiments.

This study was approved by the Ethical Committee of Tokyo Medical and Dental University (approval number: 601) and Nihon University School of Medicine (approval number: 22-3-1). All participants received an adequate explanation regarding the study rationale and procedures prior to the experiments. They all provided written informed consent and were free to withdraw from the study at any time.

### Imaging Data Acquisition

Magnetic resonance imaging was performed at Nihon University School of Medicine (Tokyo, Japan), using a 1.5T scanner (Symphony, Siemens, Erlangen, Germany) with a head coil. For each participant, we acquired a time series of whole-brain scans of the T2*-weighted gradient-echo echo-planar images with the following parameters: TR, 2000 ms; TE, 50 ms; flip angle, 90 degrees; slice thickness, 6 mm; gapless; 20 horizontal slices parallel to the AC-PC line; FOV, 192 × 192 mm; image matrix, 64 × 64; acquisition order, descending. We also obtained the T1-weighted anatomical images of 192 whole-brain sagittal scans for each participant with the following parameters: TR, 2200 ms; TE, 3.93 ms; flip angle, 15 degrees; slice thickness, 1 mm; FOV, 256 × 224 mm; in-plane resolution, 1 mm; orientation, sagittal.

### Stereomotion Localizer (SL) Session

In this scanning session, we examined cortical regions activated by the motion of a square toward and away from the observer represented by a binocular disparity cue. We termed these activated regions SMRs, and defined the whole activation area as the functional regions of interest (ROIs). See the Imaging Data Analysis section for details.

#### Visual Stimulus

In this session, all stimuli were presented binocularly via MR-compatible fiber optic glasses (SV-4021, Avotec, Inc., Stuart, FL, USA) attached to the head coil. The spatial resolution of the glasses was 480 × 640 pixels (height × width).

We used software for three-dimensional drawing (Omega Space ver. 2.1, Solidray Co. Ltd., Yokohama, Japan) to create a random dot stereogram (RDS). Small red dots of 2 × 2 pixels were randomly distributed over a black background. The dots occupied 65% of the stimulus, and their distribution was changed randomly from trial to trial. We employed three conditions:

1. Moving square (mSQ) condition: A square (7.1 × 7.1 visual degrees in height and width) was presented in the center of the background (16.8 × 22.4 visual degrees) (Fig 1B). The binocular disparity of the background was 0. The square moved toward and away from the observer two times in 2 s. Three sub-conditions were designed: mSQ1, mSQ2, and mSQ3. The binocular disparity was changed from 0 to 0.396, 0.930, and 1.402 degrees over time so that the simulated distance of the square from the background (indicated by *d* in Fig 1B) became 1, 2.5, and 4 cm at the foremost position in the mSQ1, mSQ2, and mSQ3 sub-conditions, respectively. The velocity of the square was sinusoidally modulated, and the size of the retinal image of the square was kept constant during the motion. At the end of the stimulus presentation period, the square disappeared.
2. Static square (sSQ) condition: The square was presented in front of the background for 2 s. Three sub-conditions were designed: sSQ1, sSQ2, and sSQ3. The simulated distance between the square and background in sSQ1, sSQ2, and sSQ3 was same as the maximum distances in mSQ1, mSQ2, and mSQ3, respectively. The size of the retinal image of the square in sSQ1, sSQ2, and sSQ3 was same as that in mSQ1, mSQ2, and mSQ3 at the foremost position, respectively. At the end of the stimulus presentation period, the square disappeared.
3. The no square (nSQ) condition: Only the background was presented for 2 s. There were no sub-conditions.

#### Task

fMRI data were collected by event-related design. A scanning session started with an initial rest period of 20 s. In a trial, the visual stimulus was presented for 2 s, followed by an inter-trial interval (ITI) of 1–8 s (mean: 4 s). The stimuli of the mSQ, sSQ, and nSQ conditions were presented 72 times in pseudo-randomized order during a scanning session (each of the three sub-conditions of the mSQ and sSQ conditions was presented 24 times). The session ended with a final 20-s rest period. A fixation target (a yellow cross of 1 visual degree in height and width) was presented at the center of the background during the ITI and the rest periods. No fixation target was presented during the trials, and the participants were asked to look at the center of the square (see S1 Text for details on the eye movements). To ensure that the participants perceived depth in the stimuli, they were required to press a button with one hand when they perceived the square as moving or in depth (positive response), and with the opposite hand when they did not observe any motion or depth in the stimulus (negative response). The hands corresponding to positive and negative responses were balanced across participants. We calculated the incidence of positive responses for each of the three conditions, and averaged across participants (response ratio). Trials with no response were omitted from analysis. The behavioral results for the SL session are described in S2 Text and S1 Table.

### Cast Shadow (CS) Session

#### Visual Stimulus

The visual stimulus was projected onto the screen attached to the top of the head coil, and the participants observed it through a tilted mirror. The distance between the center of the mirror and the eyes of the participants was about 22 cm. All visual stimuli were presented using E-Prime^®^ v. 2.0 (Psychology Software Tools, Inc., Sharpsburg, PA, USA). There was no binocular disparity in the stimuli in this session.

We prepared movies that were modified from the original SOC illusion. All movies were created using drawing software (Illustrator and Photoshop CS4, Adobe Systems Incorporated, San Jose, CA, USA) and custom programs written in Matlab (MathWorks, Natick, MA, USA). A green square was presented at the center of a blue-white checkerboard background. The sizes of the square and background were the same as those in the SL session. Three conditions were employed, as follows:

1. The moving cast shadow (mCS) condition: A cast shadow was presented at the bottom-right corner of the central square (Fig 1A, top section). The cast shadow moved away from and toward the square two times on an oblique rightward trajectory during the 2-s presentation period. The velocity of the cast shadow was sinusoidally modulated. As the shadow moved toward the right-most position, the transparency and blurring of its border increased. We designed three sub-conditions: mCS1, mCS2, and mCS3 (Fig 1A, top section). The maximum displacement of the cast shadow from the square (*d* in the inset of Fig 1A) was 0.6, 1.4, and 2.1 visual degrees, respectively. The position, blur, and transparency of the cast shadow were not based precisely on optics, but were designed by the experimenters to create the realistic appearance of a shadow.
2. The scrambled cast shadow (sCS) condition: In this condition, the cast shadow was divided into small compartments, and the temporal order of the compartments was scrambled while the spatial position remained constant (see S3 Text and S1 Fig for details). The scrambled cast shadow was presented for 2 s. Three sub-conditions, sCS1, sCS2, and sCS3, were employed (Fig 1A, middle section). The area of the scrambled shadow in sCS1, sCS2, and sCS3 was identical to that swept by the cast shadow in mCS1, mCS2, and mCS3, respectively.
3. The no cast shadow (nCS) condition: no cast shadow was presented in the stimulus, and the square and background were same as in the mCS and sCS conditions (Fig 1A, bottom left). This condition was designed as the ‘lower-level’ control for the mCS and sCS conditions. There were no sub-conditions.

#### Task

The time sequence of the stimulus presentation was the same as that in the SL session. The stimuli of the mCS, sCS, and nCS conditions were presented 72 times in pseudo-randomized order during a scanning session (each of the three sub-conditions of the mCS and sCS conditions was presented 24 times). Participants performed the same discrimination task as in the SL session. The behavioral results for the CS session are shown in S2 Text and S1 Table.

### Imaging Data Analysis

Data were analyzed using the SPM8 software package (Wellcome Trust Centre for Neuroimaging. Available at: http://www.fil.ion.ucl.ac.uk/spm/. Accessed 15 July 2016). Volumes for the initial rest period were discarded. To ensure optimal estimation in the co-register and normalization of preprocessing, the anatomical and functional images were reoriented manually so that the origin of the images was on the anterior commissure. After motion correction and slice timing correction, each anatomical image was co-registered to the mean functional image of a session. All functional images of each participant were normalized to the Montreal Neurological Institute (MNI) brain template. Finally, the functional images were spatially smoothed via an 8-mm isotropic FWHM Gaussian kernel.

We constructed a separate general linear model (GLM) for each participant. Data from the SL and CS sessions were analyzed separately. In the SL session, the sub-conditions mSQ1, mSQ2, and mSQ3 were modeled as part of the mSQ condition, and the sSQ1, sSQ2, and sSQ3 were modeled as part of the sSQ condition. Similarly, the sub-conditions mCS1, mCS2, and mCS3, and sCS1, sCS2, and sCS3 were merged into conditions mCS and sCS. Thus, the model was constructed with three conditions for the SL (mSQ, sSQ, and nSQ) and CS (mCS, sCS, and nCS) sessions. At the model estimation stage, the MRI data were high-pass filtered with a cutoff of 128 s to remove low-frequency drifts. Global scaling was not applied to the data. For the SL session, we investigated the cortical regions that selectively responded to the motion in depth represented by binocular disparity according to the following contrast: mSQ > (sSQ + nSQ). In the CS session, we investigated two contrast images: mCS > nCS and sCS > nCS.

Contrast images of the conditions were entered into a second-level random-effects analysis using a one-sample t-test on a voxel-by-voxel basis. We applied a statistical threshold of p < 1.0 × 10^−5^, uncorrected for multiple comparisons. Only clusters of more than 50 voxels were analyzed. The coordinates of the activations were converted from MNI to Talairach coordinates using a non-linear transform [25]. The locations of the activations were identified using a human brain atlas [26] and an anatomical toolbox for SPM8 (SPM Anatomy Toolbox v. 2.0) [27].

The activations obtained by the contrast mSQ > (sSQ + nSQ) in the SL session were designated as the stereomotion regions (SMRs). Functional ROIs were defined in each of the whole activation areas of these regions. We investigated the percent signal change in the BOLD signal of the SMRs in the SL and CS sessions, using a toolbox for ROI analysis in SPM8 (MarsBaR v. 0.44) [28]. Although we defined the functional ROIs using data from the SL session, we applied the ROI analysis to the same session to compare the activity between hemispheres.

### Distance Estimation Test

To elucidate whether the motion in depth induced by binocular disparity and by moving cast shadow were perceptually equivalent, we conducted a distance estimation test after the scanning sessions. A physical test board with a square plate that was movable in depth (Fig 1C) was prepared (see also S4 Text for details on the test board). The board was placed on a desk so that the size of the retinal images of the plate was same as that of the central square in the stimuli presented in the SL and CS sessions. Participants sat at a desk with their chin on a chin rest. In the test for the stimuli presented in the SL session, participants wore a head-mounted display (spatial resolution: 480 × 640 pixels in height and width). In each trial, one of the seven stimuli from the SL session was presented with binocular disparity. After the stimulus presentation, the experimenter switched the head-mounted display to the transparent mode so that the participant could see the test board through the display. Participants were asked to adjust the position of the plate on the test board so that it matched the perceived foremost position of the square in the stimulus they had observed (Fig 1C). In the test for the stimuli presented in the CS session, the visual stimulus was presented on a flat monitor placed beside the test board. The position of the monitor was such that the sizes of the retinal image of the square presented on the monitor and that of the plate on the test board were the same. Each stimulus was presented three times in pseudo-randomized order. The position of the plate adjusted by participants was measured by an experimenter (see S4 Text for details). When participants did not perceive any depth in the stimulus, they were asked to report this orally.

### Statistical Analysis

The data from the ROI analysis and the distance estimation test were entered into a repeated-measures analysis of variance (ANOVA). A post hoc test was performed by Ryan’s method. All significance levels were set at p < 0.05.

## Results

### Results of the Distance Estimation Test

**Fig 2:**
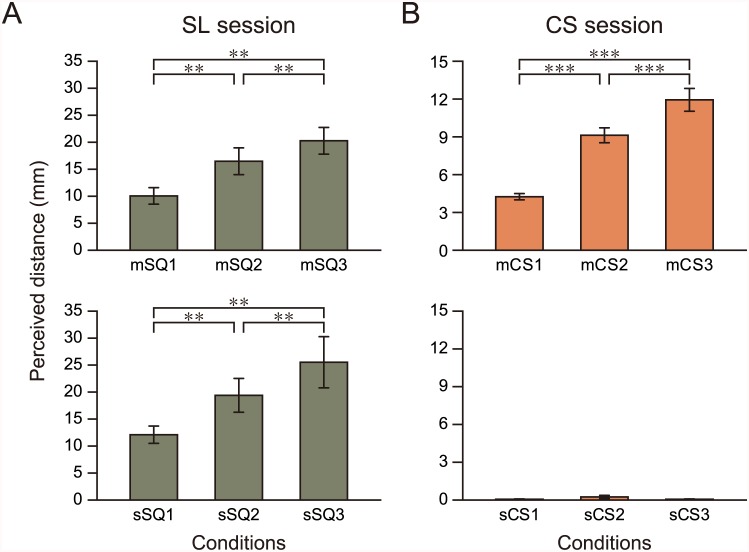
Results of the distance estimation test. The perceived distances between the square and background for the visual stimuli in the SL (A) and CS (B) sessions are shown. The measured distances were averaged across all participants. Error bars: S.E.; **: *p* < 0.01; ***: *p* < 0.001.

Fig 2 shows the results of the test. When participants observed the stimuli in the mSQ condition during the SL session, the perceived distance between the square and background increased as the maximum binocular disparity increased from mSQ1 to mSQ3 (Fig 2A, top). Similarly, in the sSQ condition, the perceived distance increased as the maximum binocular disparity increased from sSQ1 to sSQ3 (Fig 2A, bottom). We performed a two-way repeated measures ANOVA to assess the main effects of the motion of the square (moving [mSQ] vs. static [sSQ]) and the sub-conditions (mSQ1 vs. mSQ2 vs. mSQ3 and sSQ1 vs. sSQ2 vs. sSQ3 in the mSQ and sSQ conditions, respectively). The nSQ condition was omitted from the analysis, because no participant perceived any depth in this condition. The statistical analysis indicated a significant main effect of both motion (*F*_1, 20_ = 9.51, *p* < 0.01) and sub-condition (*F*_2, 40_ = 21.58, *p* < 0.001), and no interaction of these variables (*F*_2, 40_ = 1.32, *p* = 0.28). A post hoc test indicated a significantly larger perceived distance in the sSQ condition compared with the mSQ condition, and a significant difference between all pairs of the sub-conditions (*p* < 0.01 for all comparisons).

In the CS session, the perceived distance between the square and background gradually increased as the maximum displacement of the cast shadow from the square increased from mCS1 to mCS3 (Fig 2B, top). However, almost no participant perceived the motion or distance of the square in depth in the sCS condition (Fig 2B, bottom). No participant perceived any depth in the nCS condition; therefore, this condition was excluded from further analysis. Significant main effects emerged for the state of the cast shadow (moving [mCS] vs. scrambled cast shadow [sCS], *F*_1, 20_ = 246.30, *p* < 0.001) and for sub-condition (*F*_2, 40_ = 76.68, *p* < 0.001), and the interaction was also significant (*F*_2, 40_ = 71.78, *p* < 0.001). A post hoc test revealed a significant difference between all pairs of the sub-conditions in the mCS condition (*p* < 0.001 for all comparisons), but not in the sCS condition (the *p* value for the main effect on the sCS condition was 0.92).

The relationship of the perceived distance between the mSQ and mCS conditions was investigated at the individual level. The average Pearson’s correlation coefficient for all participants was 0.94 ± 0.07 (mean ± S.D.), indicating a strong relationship between the two conditions. The correlation was significant in 12 of the 21 participants at *p* < 0.05 (the *p* values averaged over the 12 and over all 21 participants were 0.02 ± 0.01 and 0.06 ± 0.07, respectively). The mean of the slope of the regression line was 0.73 ± 0.45, suggesting that the perceived distance was slightly smaller in the mCS condition than in the mSQ condition.

The results of the distance estimation test indicate that the visual stimuli presented in the SL and CS sessions could successfully evoke motion perception in depth in the participants, and the evoked motions were perceptually comparable despite the differences in the physical properties of the depth cues in the two sessions. These results also provide a rationale for using the stimuli in the mSQ condition to define the SMR for the ROI analysis in the fMRI experiment.

### Results of the fMRI Experiment 1: ROI Analysis

**Fig 3:**
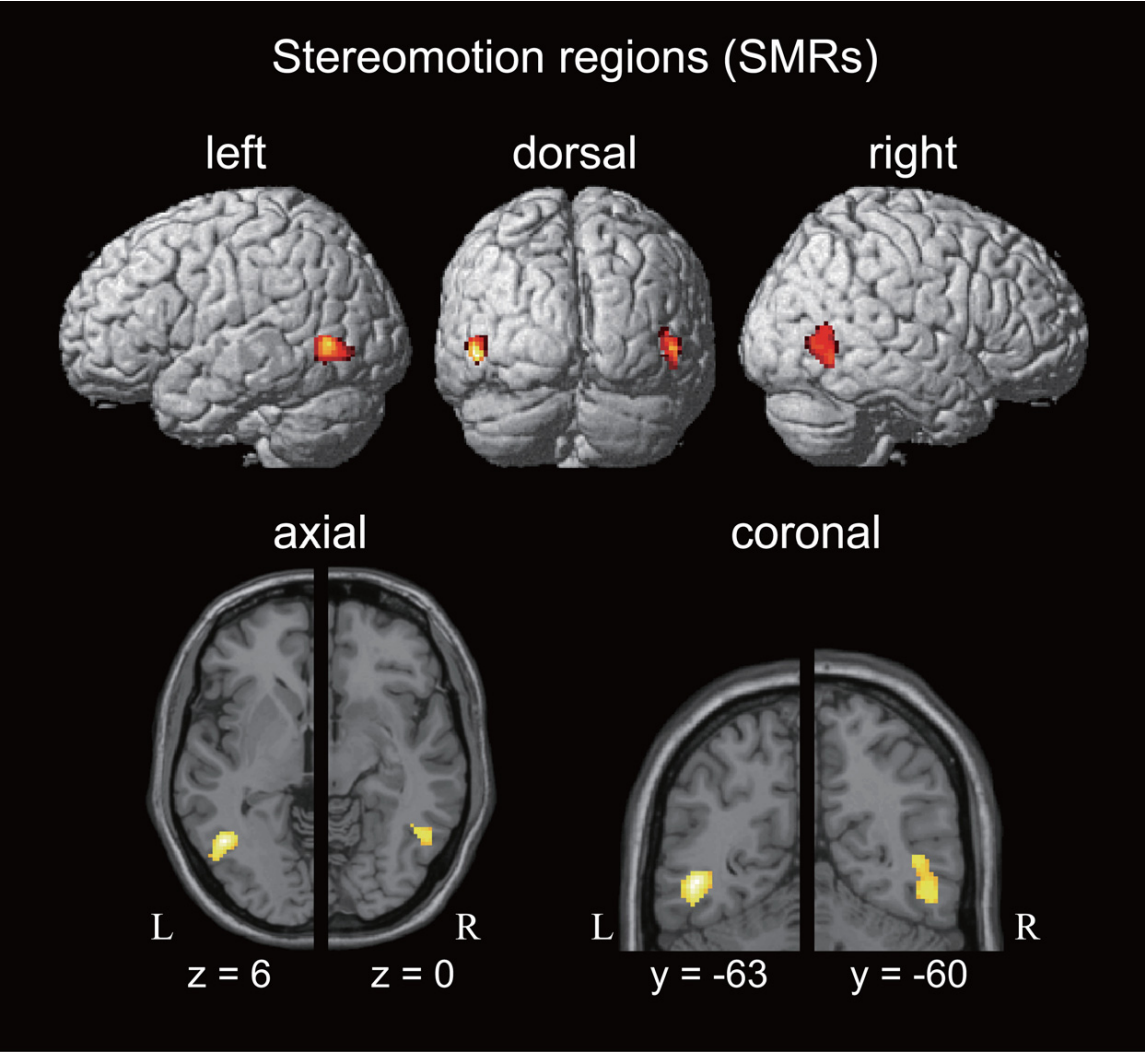
Stereomotion regions (SMRs). Top section: activation obtained by the contrast mSQ > (sSQ + nSQ) in the SL session, rendered on the cortical surface. Lower section: axial (left) and coronal (right) sections of the SMRs at the peak voxel in both hemispheres. The functional ROIs were defined on the whole activation area of the regions.

To investigate the cortical regions involved in motion perception in depth represented by the binocular disparity cue, we investigated the contrast image of mSQ > (sSQ + nSQ) in the SL session. We observed significant bilateral activation in the posterior portion of the inferior temporal sulcus (ITS) (Fig 3), and designated these areas as SMRs. According to a statistical anatomical map [27], the SMR was located anterior to the hMT, and the overlap of these two areas was 15.9% and 18.2% in the left and right hemispheres, respectively. The functional ROI was defined as the entire SMR in the left and right hemispheres (Table 1).

**Table 1:** SMRs obtained in the SL session.

Location	Talairach coordinates			Z value	Cluster size (voxel)
	x	y	z		
L. inferior temporal sulcus	−45	−63	6	5.46	258
R. inferior temporal sulcus	48	−60	0	4.82	264

Anatomical location, Talairach coordinates, Z values at the peak voxel, and cluster sizes of the SMRs obtained by the contrast of mSQ > (sSQ + nSQ) in the SL session are shown. L: left hemisphere; R: right hemisphere.

**Fig 4:**
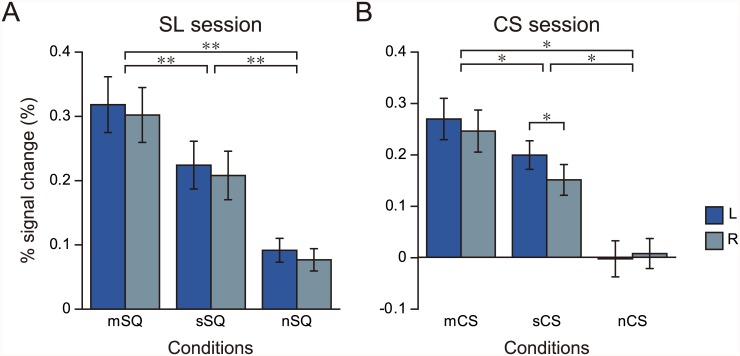
Results of the ROI analysis. The % signal change of the SMRs, averaged across all participants during observation of stimulus in the SL (A) and CS (B) sessions. Error bars: S. E.; L: left hemisphere; R: right hemisphere; *: *p* < 0.01; **: *p* < 0.001.

Next, we investigated the activity in the SMRs during the SL and CS sessions. Fig 4A shows the percent signal change of the region in both hemispheres during the SL session. We performed a two-way repeated measures ANOVA for the main effects of condition (mSQ vs. sSQ vs. nSQ) and laterality (left vs. right). The analysis showed a significant main effect of condition (*F*_2, 40_ = 36.97, *p* < 0.001), but not of laterality (*F*_1, 20_ = 1.71, *p* = 0.21). No interaction was observed between these variables (*F*_2, 40_ = 0.02, *p* = 0.99). A post hoc test showed that activity in the mSQ was significantly higher than that in the sSQ and nSQ conditions in both hemispheres (*p* < 0.001). The difference in activation between the sSQ and nSQ conditions was also significant (*p* < 0.001 for both hemispheres).

Fig 4B shows the percent signal change in the SMRs during the CS session. A two-way repeated measures ANOVA for the main effects of condition (mCS vs. sCS vs. nCS) and laterality (left vs. right) revealed a significant main effect of condition (*F*_2, 40_ = 45.19, *p* < 0.001), but not of laterality (*F*_1, 20_ = 2.78, *p* = 0.11). The interaction was also significant (*F*_2, 40_ = 3.88, *p* < 0.05). A post hoc test indicated that the activation was significantly larger in the mCS than in the sCS condition (*p* < 0.01) and the nCS condition (*p* < 0.001) in both hemispheres. The difference in activation between the sCS and nCS conditions was also significant (*p* < 0.001 for both hemispheres). In the mCS condition, we found no laterality difference in the activation (*p* = 0.19), whereas the activation of the left hemisphere was significantly larger than that of the right in the sCS condition (*p* < 0.01). There was no laterality difference in the activation of the nCS condition, either (*p* = 0.55). The moving cast shadow and the scrambled shadow appeared mainly in the bottom-right quadrant of the visual field in the mCS and sCS conditions. To quantify the physical difference in the visual stimuli presented in the left and right visual hemifields, we calculated a dissimilarity index. The index enabled us to find the mean point-wise difference between the neighboring movie frames of the stimuli (see S5 Text for details). The calculated indices for the mCS and sCS conditions are shown in Table 2. In the mCS condition, a two-way repeated measures ANOVA showed a significant main effect for sub-condition (*F*_2, 212_ = 104.22, *p* < 0.0001) and for laterality (*F*_1, 106_ = 111.35, *p* < 0.001), as well as for the interaction (*F*_2, 212_ = 108.16, *p* < 0.0001). A post hoc test indicated that the index was significantly larger in the right visual field compared with the left for all sub-conditions (*p* < 0.001). Specifically, the index was about 1.6 times larger in the right visual field. Similarly, the analysis for the sCS condition revealed a significant main effect for sub-condition (*F*_2, 212_ = 473.30, *p* < 0.001) and for laterality (*F*_1, 106_ = 1345.55, *p* < 0.001), as well as for the interaction (*F*_2, 212_ = 57.95, *p* < 0.001). A post hoc test showed that the index was significantly larger in the right visual field than in the left for all sub-conditions (*p* < 0.001), and the mean ratio of the index between the hemispheres was 1.8. These results indicate that the dissimilarity was significantly larger in the right than in the left visual hemifield in both mCS and sCS conditions.

**Table 2:** Dissimilarity index of the visual stimuli in the CS session.

×10^−3^	Left	Right	Right/Left
mCS Condition			
mCS1	1.5 ± 0.1	2.4 ± 0.2	1.60
mCS2	2.4 ± 0.2	3.8 ± 0.3	1.57
mCS3	2.9 ± 0.2	4.8 ± 0.4	1.62
mean	2.3 ± 0.4	3.7 ± 0.7	**1.60**
sCS Condition			
sCS1	3.9 ± 0.1	6.9 ± 0.1	1.76
sCS2	5.8 ± 0.2	10.6 ± 0.1	1.84
sCS3	7.0 ± 0.1	13.4 ± 0.2	1.91
mean	5.6 ± 0.9	10.3 ± 1.9	**1.84**

The dissimilarity index of the stimuli presented in the left and right visual hemifields in the mCS and sCS conditions. The indexes averaged across all pairs of the movie frames (107 pairs) for each sub-condition are shown. The right column indicates the ratio of the indices of the right and left visual hemifields.

### Results of the fMRI Experiment 2: Direct Comparison

**Fig 5:**
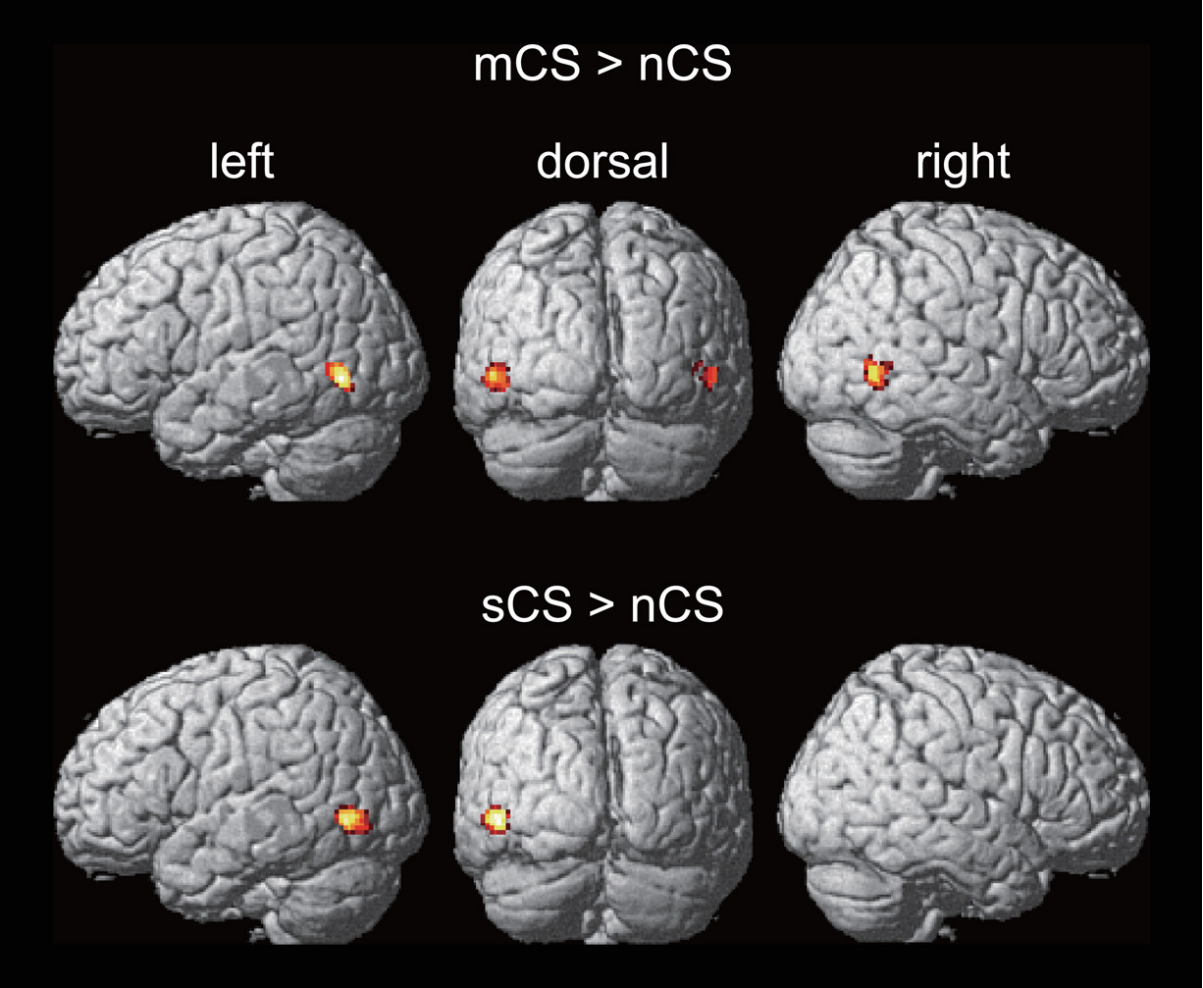
Results of the direct comparison. Activated regions revealed by contrasts mCS > nCS and sCS > nCS in the CS session are shown in the top and bottom sections of the figure, respectively.

Finally, we examined the cortical activity during the CS session by direct comparison. By the contrast of mCS > nCS, we found significant activation in the posterior portion of the ITS bilaterally (Fig 5, top section). The overlap between the activation and the statistical anatomical map of the hMT was 10.5% and 1.2% in the left and right hemispheres, respectively. In contrast, the activation was more overlapped with the SMR, and its peak coordinates were very close in both hemispheres (Tables 1 and 3). However, the contrast of sCS > nCS revealed a significant activation in the anterior occipital sulcus in the left hemisphere, and no activation in the right hemisphere (Fig 5, bottom section). The overlap with the statistical anatomical map of the hMT was 27.1%. The peak of the activation was located posterior to that of the SMR in the left hemisphere (Tables 1 and 3).

**Table 3:** Activated regions obtained in the CS session.

Location	Talairach coordinates			Z value	Cluster size (voxel)
	x	y	z		
mCS > nCS					
L. inferior temporal sulcus	−51	−66	5	5.38	153
R. inferior temporal sulcus	48	-59	6	5.01	156
sCS > nCS					
L. anterior occipital sulcus	−51	−70	1	5.31	168

Anatomical location, Talairach coordinates, Z values at the peak voxel, and cluster sizes of the activated regions obtained by the contrast of mCS > nCS and sCS > nCS are shown.

## Discussion

In the present study, we used an fMRI experiment to examine the possibility that the hMT+ is involved in motion perception in depth induced by a moving cast shadow. For this purpose, we conducted ROI analysis. We first determined the cortical regions activated by the motion of a square toward and away from the observer represented via binocular disparity in the SL session. In accordance with previous studies, we found bilateral activations in the hMT+. We designated such activations as the SMRs, and defined functional ROIs on them. We then examined the activity of the SMRs during observation of the stimuli in the CS session. In the distance estimation test, participants reported that they perceived the motion of the square in depth in the mCS condition but not in the sCS and nCS conditions. The ROI analysis showed that activation in the SMRs was significantly higher in the mCS condition compared with that in the sCS and nCS conditions. We also found that the SMR was activated equally in both hemispheres in the mCS condition, although the dissimilarity index of the visual stimuli was 1.6 times larger in the right visual hemifield than in the left. In the sCS condition, the dissimilarity index was 1.8 times larger in the right visual hemifield compared with the left, and the activity in the SMR was significantly larger in the left hemisphere. Because the bilateral activation of the SMR was also observed in the mSQ condition of the SL session, these observations suggest that the SMR may be involved in the perception of the square’s movement in depth at the center of the visual field induced by both binocular disparity and the moving cast shadow.

### Motion Perception in Depth by a Moving Cast Shadow and Binocular Disparity

The perceived distance between the square and background during observation of the visual stimuli was investigated with the distance estimation test. When participants observed the stimuli in the mCS condition, the perceived distance significantly increased as the maximum displacement of the cast shadow increased from mCS1 to mCS3. In the post-experimental interview, no participants reported feelings of unreality or curiosity regarding the stimuli during the experiments, despite the fact that the spatial configuration of the square and cast shadow was not precisely based on optics. These results indicate that the moving cast shadow in the mCS condition successfully induced the perception of the square moving toward and away from the observer.

The perceived distance obtained in the mCS and mSQ conditions showed a good linear correlation. Because the moving cast shadow and binocular disparity were the only depth cues in the mCS and mSQ condition, respectively, the result indicates that the motion in depth induced by the two depth cues was perceptually comparable. This is in line with a previous study by Khuu et al. (2014) that investigated the relationship between motion in depth induced by binocular disparity and by a moving cast shadow. The study compared the perceived speed of a central square during observation of stimuli with either the disparity or the shadow, but not both, and found that the perceived speed of the square in depth represented by binocular disparity correlated well with the physical velocity of the cast shadow in the 2D background.

### Activity of the Stereomotion Region in the CS Condition

We investigated the activity of the SMRs in the CS session. The ROI analysis showed that the activity in the SMRs was highest in the mCS condition, followed by the sCS and nCS conditions. This observation indicates that the SMRs may also respond to motion in depth induced by a moving cast shadow.

However, one may argue that the activity of the SMRs is simply a reflection of the physical differences in the visual stimuli in the three conditions. The stimuli in the mCS condition contained blurred contrast edges moving vertically and horizontally. The stimuli in the sCS condition included many randomly blinking patches, and there were no moving or blinking objects in the nCS condition. Previous studies have shown that the hMT+ is sensitive to drifting gratings with a low contrast [2], and that moving edges can evoke stronger visual responses in the MT neurons of macaque monkeys than do blinking bars [29–32]. Therefore, the stronger activation of the SMRs in the mCS condition could be at least partly due to the presence of the moving edges.

Nevertheless, the physical differences of the visual stimuli cannot fully explain the bilateral activation of the SMR in the mCS condition. The dissimilarity index of the visual stimuli in the mCS condition was significantly larger in the right visual hemifield compared with the left (1.6 times). If the activity in the SMRs simply reflects the physical difference of the visual stimuli, this activity should be larger in the left hemisphere than the right. In fact, the dissimilarity index in the sCS condition was about 1.8 times larger in the right visual hemifield than the left, and the activity in the SMRs was significantly larger in the left hemisphere compared with the right. Furthermore, the dissimilarity index was 0 in both visual hemifields in the nCS condition, and the activity of the SMRs was equal in both hemispheres. The laterality of the visual stimulus and the activity in the SMRs were mismatched only in the mCS condition. One convincing explanation for the discrepancy is that the activity in the SMRs reflects not only the physical difference of the visual stimuli but also the perceptual experience of participants as they observed the stimuli. The SMRs were identified as cortical areas activated more strongly in the mSQ condition than in the sSQ and nSQ conditions. In the mSQ condition, participants perceived motion in depth at the center of the visual field, and the activation of the SMRs was equal in both hemispheres. Because the motion in depth induced by binocular disparity and moving cast shadow were perceptually comparable, it seems reasonable to suppose that the bilateral activation of the SMRs in the mCS condition also reflects motion perception in depth across the visual hemifields.

This view is supported by the results of the direct comparisons in the CS session. The only condition that evoked motion perception in depth in the session was mCS. Given that the square and background were constant in all of the stimuli, the comparison between mCS and nCS should reflect some, if not all, of the perception of motion in depth. Consistent with the prediction, the contrast image of mCS > nCS showed bilateral activation in the posterior ITS, whereas the dissimilarity index was larger in the right visual hemifield. Furthermore, the activation largely overlapped with the SMR in both hemispheres. In contrast, comparison of the sCS and nCS conditions showed the activation only in the left anterior occipital cortex. The activity was located slightly posterior to the SMR in the hemisphere and the coordinates were closed to those of the hMT averaged over the past 11 published studies [33] (−51, −70, 1 in the present study; −44, −70, 1 in the reference [33]).

To generate the perception of a square moving across the visual hemifields, the visual system has to integrate the rectangles presented in the left and right visual fields into a single square. This process is known as interhemispheric integration, and previous studies have revealed that the hMT+ is one of the key centers involved [34, 35]. For example, when a right-facing “Pac-man”-like stimulus is presented at the center of the visual field and oscillates about the axis going through its own center, the whole Pac-man appears to oscillate at the center of the visual field, although the motion cues are restricted to the area of the “mouth” in the right visual field. Moreover, as observers viewed this stimulus, activation was observed in the hMT+ in both hemispheres [35]. This finding indicates that the hMT+ responds to a stimulus presented in the contralateral visual hemifield when the stimulus is perceived to be a part of a unified object moving across visual hemifields, even when there is no physical change in the stimulus in the contralateral visual field. Although the dimension of motion differed (2D motion in reference [35] and 3D motion in the present study), these results support our view that the bilateral activation of the SMR in the mCS condition may reflect the perceptual experience of a unified object (square) moving back and forth at the center of the visual fields. A previous study revealed that the ipsilateral hMT+ could be activated by motion signals from the contralateral hMT+ via the corpus callosum [36]. This finding may provide an anatomical basis for interhemispheric integration in this area.

### Stereomotion Region and Subdivision of the hMT+

Functional imaging studies have shown that the hMT+ is not a uniform visual area. It is divided into two subregions with independent retinotopic organizations. The posterior subregion has a relatively small receptive field around the center of the visual field and displays strong sensitivity to 2D motion. In contrast, the anterior subregion has a large receptive field covering the contralateral visual field and responds to peripheral stimuli in both visual hemifields. The anterior subregion also responds to the inward and outward motion of optic flows as well as to 2D motion. Based on these functional differences, previous studies have suggested that the posterior and anterior subregions of the hMT+ may be homologous to areas MT and MST, respectively, in macaque monkeys [37–40]. Likova and Tyler (2007) reported that the cortical area activated by 3D motion via binocular disparity (left: −43.2, −66.1, 4.3; right: 44.3, −60.4, 3.1) was located anterior to that activated by 2D motion (left: −43.6, −74.0, 0.6; right: 45.9, −68.7, 0.3). The coordinates of the 2D motion-sensitive region were close to those of the hMT [33] mentioned previously. Furthermore, the SMRs obtained in the present study (left, −45, −63, 6; right, 48, −60, 0) were closer to the 3D motion-sensitive region reported by Likova and Tyler (2007). Although we did not examine the cortical regions sensitive to 2D motion in the present study, these results suggest that the SMR may represent the human analog of MST.

### Biological Significance of Motion in Depth

As mentioned in the Introduction, previous functional imaging studies with healthy participants have revealed that the hMT+ is sensitive to motion in depth represented by various monocular depth cues [15–20]. More recently, it has been shown that the hMT+ of a blindsight patient is also activated by a looming stimulus consisting of moving dot patterns, although the patient had no awareness of the motion during observation of the stimulus [41]. This finding highlights the biological significance of motion perception in depth, especially for avoidance behavior. The direct inputs to the hMT+ from subcortical structures on the visual pathway, such as the lateral geniculate nucleus (LGN), superior colliculus (SC), and pulvinar (PL), have been considered the most likely alternative pathway, bypassing V1 and thereby accounting for the residual vision of blindsight patients [42, 43]. It has also been shown that the motion of the square in depth in the SOC illusion can be perceived even when the motion of the cast shadow is masked and processed unconsciously [24]. These observations suggest that there may be different visual pathways for processing monocular depth cues with and without consciousness, and that the hMT+ may be a key area for integrated motion perception in depth produced by those cues. The nuances of processing monocular depth cues are a problem for future study.

### Neuronal Mechanisms of Motion Perception in Depth by Moving Cast Shadow

In the present study, we showed that the hMT+ responds to motion in depth induced by both binocular disparity and a moving cast shadow. Our result points to two possibilities regarding the underlying neuronal mechanisms: one is that the motion in depth induced by a moving cast shadow and by binocular disparity are encoded by a single population of neurons; the other is that the motion elicited by the two depth cues is processed by different populations of neurons in the hMT+. If the former is the case, then motion in depth is likely represented in the activity of single neurons in a cue-independent manner. Alternatively, if the latter is true, then motion information elicited by different depth cues may be processed in parallel, even in higher visual areas. Given the limited spatial resolution of currently available functional imaging techniques, it is difficult to determine which hypothesis (or combination of them) is more plausible. The most direct evidence for addressing this question will be obtained by recording neuronal activity in the monkey visual cortex. Recent physiological studies have found that many MT neurons in macaque monkeys have direction selectivity of motion toward and away from the animal [44, 45]. These neurons are driven by binocular depth cues, such as interocular velocity differences and disparity changes over time. It is unknown whether these MT neurons also respond to motion in depth induced by monocular depth cues. Neurons in area MSTl (lateral subdivision of MST) of macaque monkeys have disparity selectivity at the center of their receptive field, and the response is enhanced by a motion stimulus presented in the surround of the receptive field [46, 47]. This center-surround interaction of the receptive field in MSTl neurons may be appropriate for processing the motion of the central square in depth induced by the cast shadow moving beside it. The involvement of the MSTl neurons in perception of motion in depth by moving cast shadow is consistent with the present result, suggesting that the SMR may correspond to the hMST, as mentioned earlier.

Recently, we have shown that macaque monkeys can also perceive motion in depth provided by a moving cast shadow. We trained a macaque monkey to perform a discrimination task with motion in depth produced by a binocular disparity cue. The moving cast shadow was presented in catch trials in which binocular disparity was not available as a depth cue because it was either too small or static. The monkey produced the same responses in the catch trials as in the trials with the binocular disparity cue, indicating that it was able to discriminate the motion direction in depth based on the 2D motion of the cast shadow [48]. This finding will enable us to conduct future investigations of the detailed neuronal mechanisms underlying motion perception in three-dimensional space.

## Supporting Information

S1 Fig Visual stimuli for the CS session (p. 13, l. 7). (TIF)

S1 Table Response ratio for the discrimination task during scanning (p. 10, l. 16 and p. 14, l. 3). (DOCX)

S1 Text Eye Movements During Scanning Sessions (p. 10, l. 9). (DOCX)

S2 Text Behavioral Results During the Scanning Sessions (p. 10, l. 16 and p. 14, l. 3). (DOCX)

S3 Text Visual stimuli for the CS session (p. 13, l. 7). (DOCX)

S4 Text Test Board for the Distance Estimation Test (p. 16, l. 10 and p. 17, l. 6). (DOCX)

S5 Text Physical Quantity of the Visual Stimuli in the CS Session (p. 23, l. 4). (DOCX)
