# Supplementary figures and images for: Activation of the Human MT Complex by Motion in Depth Induced by a Moving Cast Shadow
Source: PLoS One. 2016 Sep 6;11(9):e0162555. doi: 10.1371/journal.pone.0162555 (PMC5012579; doi:10.1371/journal.pone.0162555)

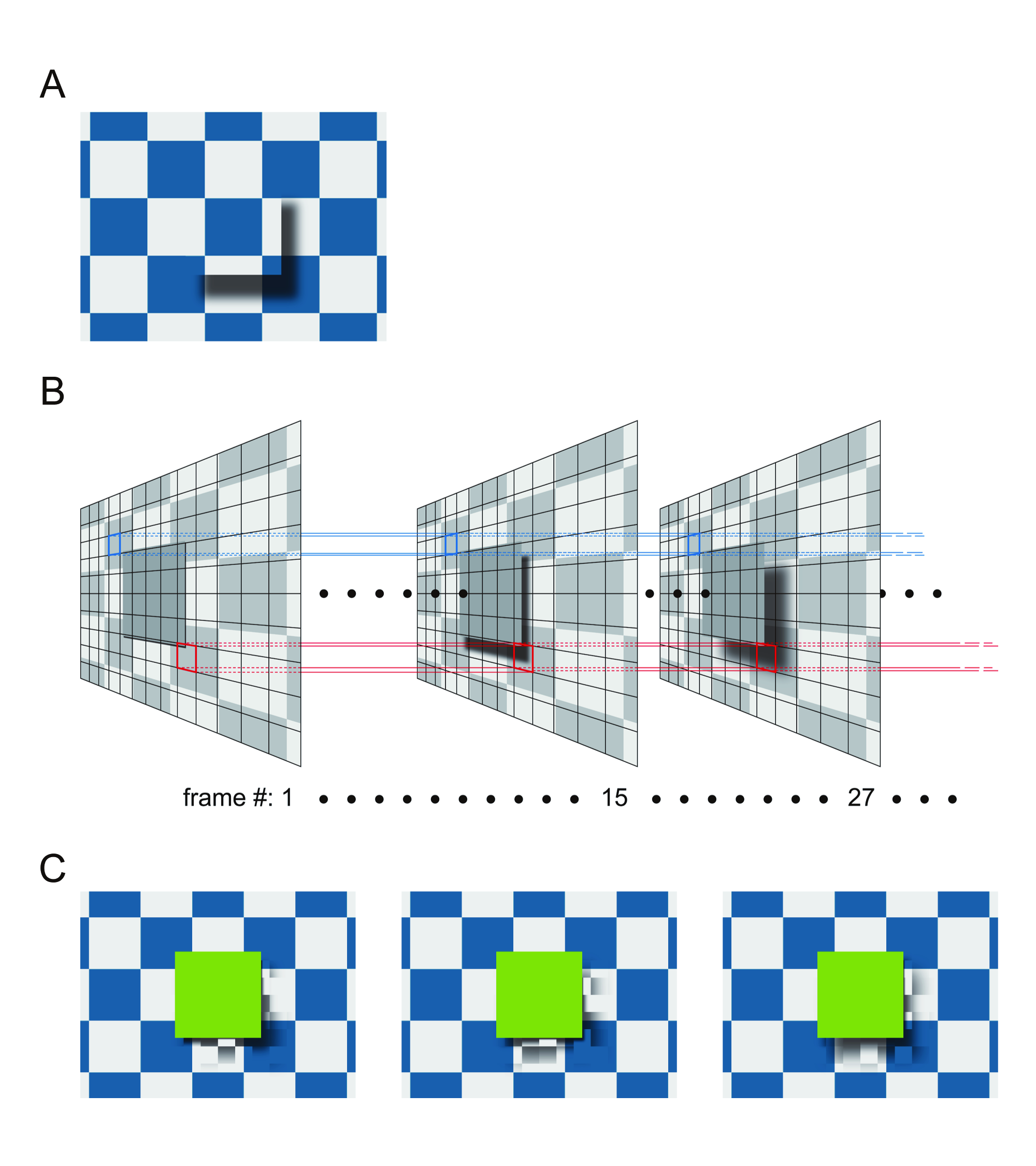

Supplement: S1 Fig — (TIF) [file pone.0162555.s001.tif]
