# Supplementary material for: Activation of the Human MT Complex by Motion in Depth Induced by a Moving Cast Shadow
Source: PLoS One. 2016 Sep 6;11(9):e0162555. doi: 10.1371/journal.pone.0162555 (PMC5012579; doi:10.1371/journal.pone.0162555)
Supplement: S1 Table — (DOCX) [file pone.0162555.s002.docx]

**S1 Table. Response ratio for the discrimination task during scanning.**

| **Session/Condition** | **Response ratio (%)** |
| --- | --- |
| SL session | |
| mSQ | 97.4 ± 2.8 |
| sSQ | 96.6 ± 5.5 |
| nSQ | 1.9 ± 2.3 |
| CS session | |
| mCS | 96.6 ± 6.2 |
| sCS | 2.8 ± 6.6 |
| nCS | 1.4 ± 2.0 |

The mean incidence of positive responses in the SL and CS sessions was calculated as the response ratio (mean ± S.D., averaged across participants).
