# Supplementary material for: Activation of the Human MT Complex by Motion in Depth Induced by a Moving Cast Shadow
Source: PLoS One. 2016 Sep 6;11(9):e0162555. doi: 10.1371/journal.pone.0162555 (PMC5012579; doi:10.1371/journal.pone.0162555)
Supplement: S1 Text — (DOCX) [file pone.0162555.s003.docx]

**Eye Movements During Scanning Sessions**

In the present study, we did not present fixation targets during the trials. Preliminary studies indicated that fixation to a target over the square prevented the perception of motion of the square in depth in the mSQ, sSQ and mCS conditions. We also tested stimuli in which the square and cast shadow were presented in the left or right visual hemifield with a central fixation target. However, the perception of motion of the square in depth was weaker than that elicited by the stimulus with the central square. Therefore, to maximize the perceptual experience of motion in depth, we presented the square at the center of the screen without a fixation target. Prior to the experiments, participants were carefully instructed to look at the center of the screen (and especially not to look outside the square) during trials. Participants completed between 15 and 25 practice trials, depending on their level of comfort with the experimental task, prior to data acquisition.
