# Supplementary material for: Activation of the Human MT Complex by Motion in Depth Induced by a Moving Cast Shadow
Source: PLoS One. 2016 Sep 6;11(9):e0162555. doi: 10.1371/journal.pone.0162555 (PMC5012579; doi:10.1371/journal.pone.0162555)
Supplement: S2 Text — (DOCX) [file pone.0162555.s004.docx]

**Behavioral Results During the Scanning Sessions**

During the SL and CS sessions, participants were required to push a button with one hand when they perceived the central square to be moving or presented in depth (positive response). The incidence of positive responses was defined as the response ratio (S1 Table). Trials without responses were omitted from the analysis. In the SL session, the response ratio was greater than 95% in the mSQ and sSQ conditions, and less than 5% in the nSQ condition. In the CS session, the response ratio was greater than 95% in the mCS condition, and less than 3% in the sCS and nCS conditions.
