# Supplementary material for: Activation of the Human MT Complex by Motion in Depth Induced by a Moving Cast Shadow
Source: PLoS One. 2016 Sep 6;11(9):e0162555. doi: 10.1371/journal.pone.0162555 (PMC5012579; doi:10.1371/journal.pone.0162555)
Supplement: S3 Text — (DOCX) [file pone.0162555.s005.docx]

**Visual stimuli for the CS session.**

Originally, we attempted to create a control that would selectively activate cortical regions sensitive to the 2D motion of cast shadows without motion perception in depth; we therefore tested a visual stimulus without a central square (S1 Fig A). The cast shadow (and its motion) and background were the same as those in the stimulus of the mCS condition. However, when the test stimulus was presented to observers in the preliminary experiment, they perceived a ‘subjective contour in depth’ at the border of the sharp edges of the cast shadow. When the cast shadow moved away from and toward the center of the background, the subjective contour at the border appeared to move toward and away from the observers, respectively. For this reason, we abandoned this stimulus as a control for the mCS condition.

The central square and cast shadow were in different locations in the visual field. This suggests that the cortical regions representing the 3D motion of the square and 2D motion of the cast shadow may be retinotopically different. If we can detect the cortical regions sensitive to the location of the cast shadow and compare it with those activated during observation of the stimulus in the mCS condition, we might be able to isolate the regions sensitive to 3D motion in depth. This was the motive for designing the stimulus for the sCS condition.

The schematic diagram of the generation of the stimulus in the sCS condition is illustrated in S1 Fig B. The movies in the mCS condition (mCS1, mCS2, and mCS3) comprised 108 static images (frames). First, all of the movie frames were divided into small compartments (indicated by grids), and the temporal order of the compartments was shuffled, keeping position constant. Then the shuffled frames were converted into new movies. To ensure consistency in terms of the total amount of energy projected on the retinae during stimulus presentation, the stimuli in sCS1, sCS2, and sCS3 were created using the movie frames from mCS1, mCS2, and mCS3, respectively. Of the divided compartments, some included a part of the cast shadow and others did not, depending on the position in the frame and the frame number. For example, a compartment indicated by a red rectangle is located at the bottom-right corner of the square. It contains a part of the cast shadow, and the extent to which the shadow appears in the compartment increases and decreases two times as the frame proceeds from 1 to 108. Shuffling the temporal order of the compartments created patterns of scrambled rectangles with different levels of contrast in the new stimulus. For comparison, the compartment located in the top-left quadrant of the stimulus (indicated by a blue rectangle) contains no fragment of the cast shadow in any of the movie frames. Therefore, the temporal shuffling of the compartments produced no change in this component in the new stimulus. Note that the area of the scrambled cast shadow in sCS1, sCS2, and sCS3 is the same as that swept by the cast shadow in mCS1, mCS2, and mCS3, respectively. Examples of movie frames from the stimulus in the sCS3 condition are shown in S1 Fig C.

In the preliminary experiment, we presented several test stimuli for the sCS condition, with various compartment sizes, to the observers. When the compartments were small, the scrambled cast shadow appeared to be made of gray matter, and the central square seemed to be presented in depth; i.e., it looked like a static image of the stimuli in the mCS condition (see the top section in Fig 1A of the main text). Conversely, when the compartments were large, the shape of the cast shadow was preserved in some compartments. Therefore, observers perceived the square in depth during some moments of the stimulus presentation. When the compartments were medium-sized, observers perceived no depth of the square in the stimuli. We therefore decided to use the medium-sized compartments (1.4 × 1.4 visual degrees in height × width) to create the stimuli for the sCS condition. The post-experimental interview showed that the participants initially experienced some curiosity regarding the stimuli in the sCS condition when they watched them for the first time in the practice session prior to data acquisition. However, they soon became familiar with the stimuli and had no curious or uncomfortable feelings during the CS session or the distance estimation test.
