# Supplementary material for: Activation of the Human MT Complex by Motion in Depth Induced by a Moving Cast Shadow
Source: PLoS One. 2016 Sep 6;11(9):e0162555. doi: 10.1371/journal.pone.0162555 (PMC5012579; doi:10.1371/journal.pone.0162555)
Supplement: S4 Text — (DOCX) [file pone.0162555.s006.docx]

**Test Board for the Distance Estimation Test**

A physical test board was prepared for the test (Fig 1C). A blue-white checkerboard was rendered on a board (background). A small rectangular hole (1 × 1 cm in height × width) was at the center of the background. An acrylic rod (1 × 1 × 20 cm in height × width × length) with a rectangular plate (3 × 3 cm) attached to one end was threaded through the hole so that the plate was on the checkerboard side. The rod could be moved back and forth on a trajectory perpendicular to the background. A scale was printed on the surface of the rod. After the participant had adjusted the position of the plate, an experimenter used the scale to measure the distance between the plate and background and recorded the value on a data sheet. At the end of each trial, the experimenter returned the plate to the background so that the distance between the plate and background was 0.
