# Supplementary material for: Activation of the Human MT Complex by Motion in Depth Induced by a Moving Cast Shadow
Source: PLoS One. 2016 Sep 6;11(9):e0162555. doi: 10.1371/journal.pone.0162555 (PMC5012579; doi:10.1371/journal.pone.0162555)
Supplement: S5 Text — (DOCX) [file pone.0162555.s007.docx]

**Physical Quantity of the Visual Stimuli in the CS Session**

The moving cast shadow in the mCS condition and the scrambled cast shadow in the sCS condition were the only dynamic objects in the stimuli. Because the stimuli in these conditions were geometrically asymmetric to the vertical axis of the center of the screen, we measured the physical quantity of the visual stimuli presented in the left and right visual hemifields via an index. In the stimuli in the mCS and sCS conditions, the image in each movie frame is slightly different from that in the neighboring frame. For these, we employed a modified version of the dissimilarity index used to assess physical picture similarity in previous studies (Grill-Spector K, Kushnir T, Edelman S, Avidan G, Itzchak Y, Malach R. Differential processing of objects under various viewing conditions in the human lateral occipital complex. Neuron. 1999; 24(1): 187-203). Each of the 108 still frames that made up a 2 s movie was 216 × 324 pixels in height and width. We calculated the mean point-wise Euclidean distance of the RGB values between the neighboring movie frames (*d_i_*), using the following expression:

$d_{i}$ = $\frac{1}{n}\sqrt{\sum_{x=1}^{n} \left\{ \left( R_{i}\left( x \right) - R_{i+1}\left( x \right) \right)^{2}+ \left( G_{i}\left( x \right)- G_{i+1}\left( x \right) \right)^{2}+ \left( B_{i}\left( x \right)- B_{i+1}\left( x \right) \right)^{2} \right\}}$

(*i* = 1, 2, 3, …, 107)

where *n* is the number of pixels in half of the movie frame (216 × 162 = 34992 pixels), *R_i_(x)*, *G_i_(x)*, and *B_i_(x)* are the color values of red, green, and blue (0–255), respectively, for a specific pixel in location *x* in the *i*-th movie frame. We then averaged the mean point-wise distance (*d_i_*) across all pairs of neighboring movie frames (107 pairs) and designated the result as the dissimilarity index of the stimulus. The index was calculated separately for the left and right halves of the stimuli in the mCS and sCS conditions. A larger value on the index indicated a larger change between neighboring movie frames. The indexes averaged across all frame pairs are shown in Table 2 in the main text. For the statistical analysis, the indexes of the 107 frame pairs for each of the sub-conditions were used (see the Results section in the main text).
